# Incidence of Branching Patterns Variations of the Arch in Aortic Dissection in Chinese Patients

**DOI:** 10.1097/MD.0000000000000795

**Published:** 2015-05-01

**Authors:** G. Pullas Tapia, Xiaohua Zhu, Jing Xu, Pan Liang, Gang Su, Hai Liu, Yang Liu, Liliang Shu, Shuiqi Liu, Chen Huang

**Affiliations:** From the Department of Cardiovascular Surgery, First Hospital Affiliated of Zhengzhou University, China.

## Abstract

Several authors have described anatomic variations of the aortic arch in 13% to 20% of the patients who do not have aortic disease. However, few studies have evaluated these patterns in the thoracic aortic dissection (TAD). In the authors’ knowledge, this is the first survey that specifically investigates the frequency of these variations in a broad, nonselected group of Chinese patients with aortic dissection. Furthermore, it compares this group with a group of patients without aortic disease.

The objective of this study was to define the variation frequency of the aortic arch branches pattern using the tomographic studies of 525 Chinese patients with a diagnosis of TAD. The Stanford classification was used to set the site of the initial tear of the dissection. In addition, we performed an epidemiological analysis of the aortic arch anatomic variations in TAD, and its possible implications for surgical or endovascular treatment. The general hypothesis proposal asserted that Chinese patients with dissection of the aorta have a similar incidence of variations of the aortic arch to the patients without aortic disease.

A retrospective study of cases and controls was carried out using the tomographic studies (CT) of all patients admitted to the First Affiliated Hospital of Zhengzhou University, located at Henan-China, with a confirmed diagnosis of aortic dissection from January 2012 until December 2014. The group of cases consisted of 525 patients: 374 men and 151 women, with a mean age of 52.27 years (range, 20–89). The average age of the patients with Stanford A and B aortic dissection was 49.46 and 53.67, respectively. The control group consisted of 525 unselected patients without TAD who underwent a CT scan of the chest due to other indications. This group consisted of 286 men and 239 women, with a mean age of 53.60 years (range, 18–89). All the patients with aneurysm or dissection were excluded from the control group. We performed a statistical analysis of demographic data.

The study found 7 different patterns of the aortic arch on both groups of cases and controls. Within the 525 patients with TAD were observed 85 (16.19%) anatomical variations, while the control group showed 112 variations (21.33%); *P* = 0.033. The most common anatomical variant was the bovine arch, found in 62 (11.80%) cases of TAD compared with 77 (14.66%) in the control group; *P* = 0.172. Anatomical variations were observed in 14.32% of the patients with Stanford A dissection and 17.09% of the patients with Stanford B dissection; *P* = 0.425. Patients with Stanford A dissection showed the pattern of bovine arch in 23 (13.21%) of 174 cases. In contrast, the patients with Stanford B dissection showed it in 39 (11.11%) of 351 cases; *P* = 0.481. The anatomical variant defined as vertebral artery of direct origin of the aortic arch was more frequent in the patients with Stanford B dissection (5.12%). The patients with Stanford A dissection presented this pattern in 1.14% of the cases; *P* = 0.025. This study observed an increased frequency of aortic dissection in the subgroup from 41 to 60 years old. In the subgroup from 41 to 60 years old without TAD, a greater frequency of anatomical variations were found than in the patients with TAD (20.81% vs 14.23%; *P* = 0.050). The same fashion was seen in patients older than 80 years (27.27% vs 0%; *P* = 0.030). The anatomical variations of the aortic arch with TAD occurred in 14.97% of the male patients and 19.20% of the female patients compared to 21.67% to 20.92% in the control group; *P* = 0.026 and *P* = 0.681, respectively.

The aortic arch variations were found less frequently in the TAD group than in the control group in the present Chinese series. The bovine arch was considered the variant pattern of the major frequency in the patients with TAD and the control group. The anatomical variant of 4 branches, defined as vertebral artery of direct origin of the aortic arch, was more frequent in patients with Stanford B aortic dissection than in the patients with Stanford A.

This finding might show an association between the geometry of the aortic arch and the site of onset of first intimal tear of dissection.

## INTRODUCTION

### Aortic Dissection

Aortic dissection is defined as a rupture of the internal layer of the artery, which drives the flow of blood through a false lumen, causing weakness of the wall with the subsequent risk of aneurysmal dilation or rupture. This disease is associated with symptoms of thoracic or abdominal pain and signs of arterial insufficiency of the organs affected by static or dynamic occlusion of the aortic branches.

The World Health Organization (WHO) has demonstrated that cardiovascular disease is the leading cause of death in the world. Hypertension, smoking, and air pollution due to solid fuel are 3 proven risk factors in the development of cardiovascular and cerebrovascular diseases.^1^ Aortic dissection, which is produced by joint risk factors to all cardiovascular illness, is a disease with high mortality despite early diagnosis and appropriate treatment. Its frequency is estimated at 3 to 4 cases per 100,000 persons per year.^2^ The prevalence of aortic dissection has a range between 0.2% and 0.8% in the Western series of autopsies.^3^ The prevalence of thoracic aortic dissection (TAD) in China is difficult to estimate due to a lack of postmortem studies.

Epidemiologically, aortic disease has been related to uncontrolled hypertension, pregnancy, trauma, bicuspid aortic valve, Marfan syndrome, Ehlers–Danlos syndrome, Turner syndrome, syphilis, and use of cocaine,^4^ as well as interventional or surgical procedures to the heart and aorta.^4^ However, several studies have determined that the cause of aortic dissection is multifactorial.

From a clinical point of view, the study of aortic disease does not account with investigation that joins hemodynamic, clinical, and basic research. Therefore, the pathophysiology of the aortic disease is not entirely apparent. Nowadays, there is a lack of clinic information about the relationship between the aortic arch branches pattern and the place of the first tear of dissection. Biodynamic studies have found that the hemodynamic and structural causes of the first intimal tear of aortic dissection are apparently dependent on the factors of the deformation of the arterial wall. These factors include thickness of the wall, the deformation-stress relationship, the impact of the tissue that surrounds the artery, the homogeneous material properties, and the blood pressure. Also included are the mass wave reflections from the arterial branches, the pulsatile movement of the heart, the residual stress, and the grade of fixation of the wall.^5,6^ Finally, the knowledge of the embryologic origin of the anatomical variations of the aortic arch is a very important issue in order to understand the pathophysiology of the aortic dissection.^7–10^. This study wants to give light to help understand the influence of the anatomical factor in the pathophysiology of the aortic dissection.

### Anatomical Variations of the Aortic Arch and TAD

Autopsy studies, computerized axial tomography, and conventional digital angiography images have demonstrated that the incidence of variations in the patterns of branching of the aortic arch in patients without aortic disease varies from 13% to 20%.^11–13^ Few studies have assessed these variations in the TAD in order to elucidate if these patterns could act as a risk factor for the disease. Wanamaker et al reviewed 179 cases of TAD and noted aortic arch variations in 34% of the cases, compared to 19% of the controls. The author concluded that anatomical variations of the arch may have a relationship with the proclivity for aortic dissection.^14^

The standard pattern of 3 branches is composed of the brachiocephalic trunk (common trunk of the right common carotid artery and the right subclavian artery), the left common carotid artery, and the left subclavian artery.^13^ This pattern takes place in 65% to 80% of the cases.^13,15^ The configuration named bovine arch, formed by the brachiocephalic trunk and the left common carotid artery in a common trunk, is the most common anatomical variation.^16^ This pattern is observed in 10% to 20% of the population.^17^ Other variations of the pattern of the aortic arch are observed less frequently and have been described as isolated cases.

The Stanford classification discriminates the aortic dissection in type A and B, depending on the primary site of the lesion before or after the aortic origin of the left subclavian artery.^18^ In the Stanford A dissection, the lesion affects the ascending aorta while type B dissection is generated after left subclavian artery implantation.

The current study attempts to analyze the patterns of the aortic arch that might have some impact on the pathophysiology of the disease. The study has an anatomical focus rather than clinical, so the clinical features of the patients were not assessed.

The objective of this study was to define the variation frequency of the aortic arch branches pattern using the tomographic studies of 525 Chinese patients with a diagnosis of TAD. The general hypothesis proposal asserted that the Chinese patients with dissection of the aorta have a similar incidence of variations of the aortic arch to the patients without aortic disease.

## MATERIALS AND METHODS

A retrospective study of cases and controls was carried out using the tomographic studies (CT) of all patients admitted to the First Affiliated Hospital of Zhengzhou University with confirmed diagnosis of aortic dissection from January 2012 until December 2014. The First Affiliated Hospital of Zhengzhou University is located in Zhengzhou, the capital of Henan province, with a population of 9,000,000 residents. Situated in the central-eastern area of China, Henan has an area of 167,000 km^2^ with an average population of almost 106,000,000 inhabitants. The hospital has 7000 beds; 300,000 people visit the emergency department every year; 4,600,000 patients are treated in the outpatient department, and 320,000 patients are admitted for hospitalization each year. The patients were identified from a systematic review of the database of the hospital. The study was approved by the Institutional Review Board. Informed consent was not required for this retrospective study. There were 525 revised CT scans with a diagnosis of TAD (n = 525, 374 men and 151 women). The images were analyzed by a cardiovascular surgeon and a radiologist for consensus. The mean age ± standard deviation (SD) of all TAD patients was 52.27 ± 13.68 years. The mean age ± SD of the patients with dissection type A and dissection type B was 49.46 ± 13.31 and 53.67 ± 13.67, respectively. The age range was 20 to 89 years. The unselected control group consisted of 525 patients without TAD, over 18 years old, who underwent chest CT scans for other indications. The control population was composed of 239 women and 286 men (54.47%), with a median age of 53.60 ± 15.05 (range, 18–89). All patients with dissection or aneurysm were excluded from the control group. Image interpretation was conducted in software designed for navigating in multidimensional DICOM images, OsiriX. Three-dimensional reconstruction surface of axial images to expose the variations in supraaortic arteries in the case group, and the aortic arch without dissection in the control group, was performed. The Stanford classification was utilized for distinguishing the dissection.^18^

### Variables

All the anatomical patterns of the aortic arch found in the case and control groups were considered variables. In addition, age and gender were analyzed.

### Statistical Analysis

The study data were analyzed using the SPSS 22.0 statistical package program (SPSS Corp, Chicago, IL). Every continuous variable was presented as mean ± SD. The χ^2^ test was used to determine the association between age and gender differences, dissection, and variations in the aortic arch branching pattern. A *P* value < 0.05 was considered statistically significant.

## RESULTS

### Aortic Arch Patterns

**FIGURE 1:**
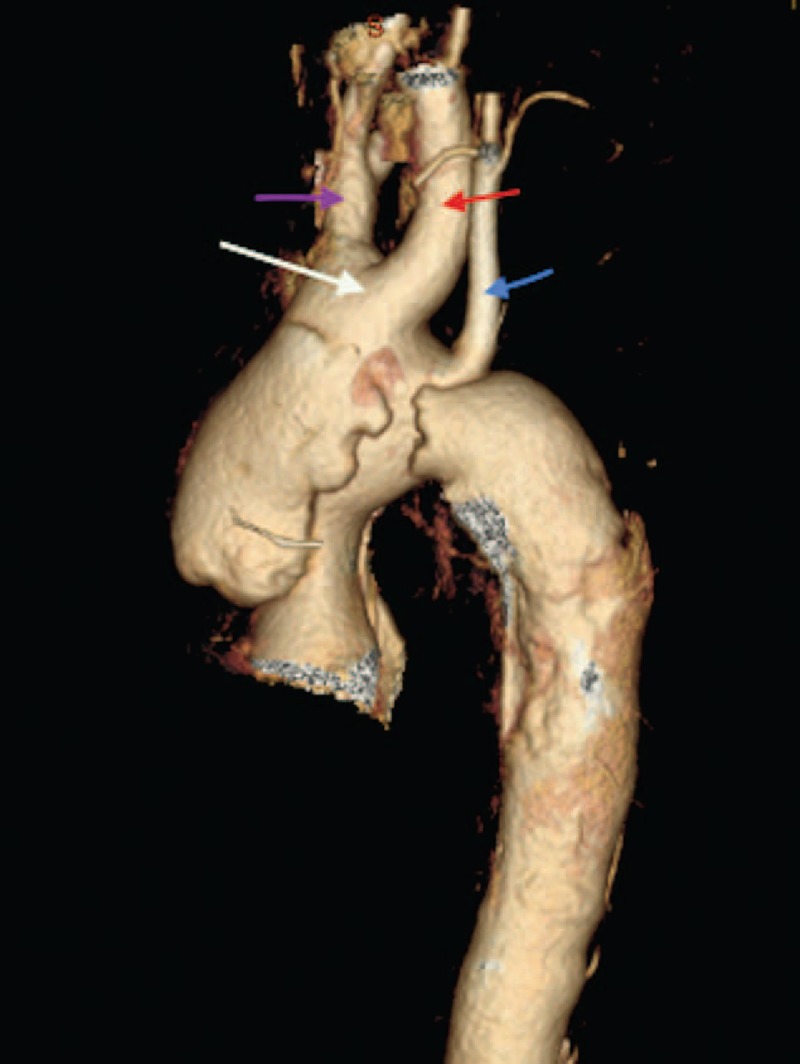
CT scan image (volume rendered). Bovine arch and aortic Stanford B aortic dissection. Brachiocephalic trunk and right common carotid artery are sharing a common root. (White arrow, bovine arch; purple arrow, brachiocephalic trunk; red arrow, left common carotid artery; blue arrow, left subclavian artery.) CT = computed tomography.

**FIGURE 2:**
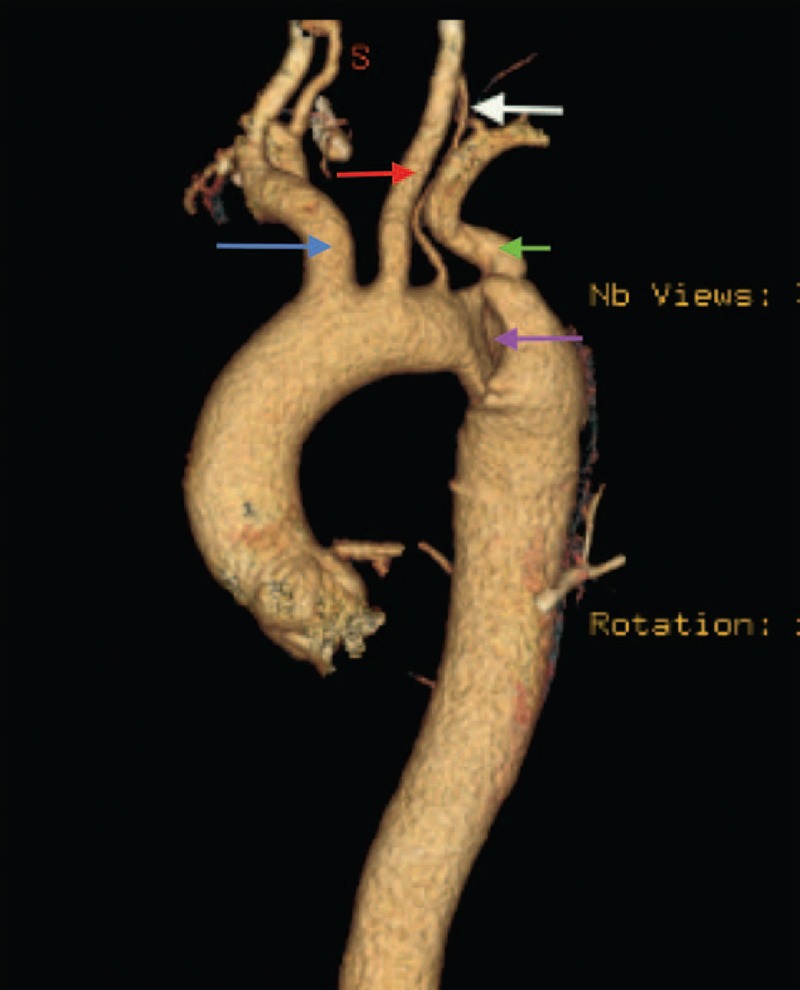
CT scan image (volume rendered). Aortic arch with vertebral pattern and Stanford B aortic dissection. (Blue arrow, brachiocephalic trunk; red arrow, left common carotid artery; white arrow, vertebral artery; green arrow, left subclavian artery; purple arrow, aortic dissection.) CT = computed tomography.

**FIGURE 3:**
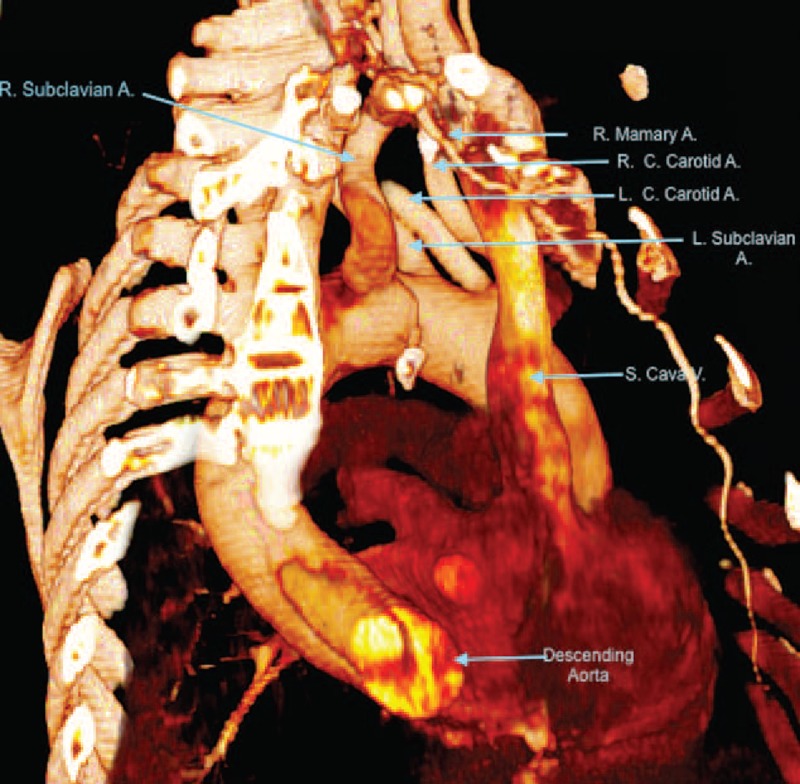
CT scan image (volume rendered). Lateral view. LARS. A = artery, CT = computed tomography, LARS = left aortic arch with right carotid artery, left carotid artery, left subclavian artery, and aberrant right subclavian artery, L.C. = left common, R = right, R.C. = right common, S = superior, V = vein.

**FIGURE 4:**
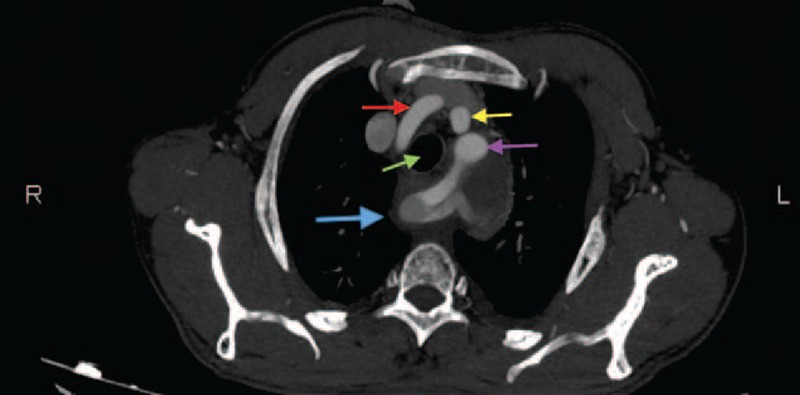
CT scan image. Axial view. LCCARS and Stanford B aortic dissection. (Blue arrow, dissected aberrant right subclavian artery; red arrow, right common carotid artery; purple arrow, left subclavian artery; yellow arrow, left common carotid artery; green arrow, trachea.) CT = computed tomography, LCCARS = left aortic arch with common origin of the carotid arteries, left subclavian artery, and aberrant right subclavian artery.

**FIGURE 5:**
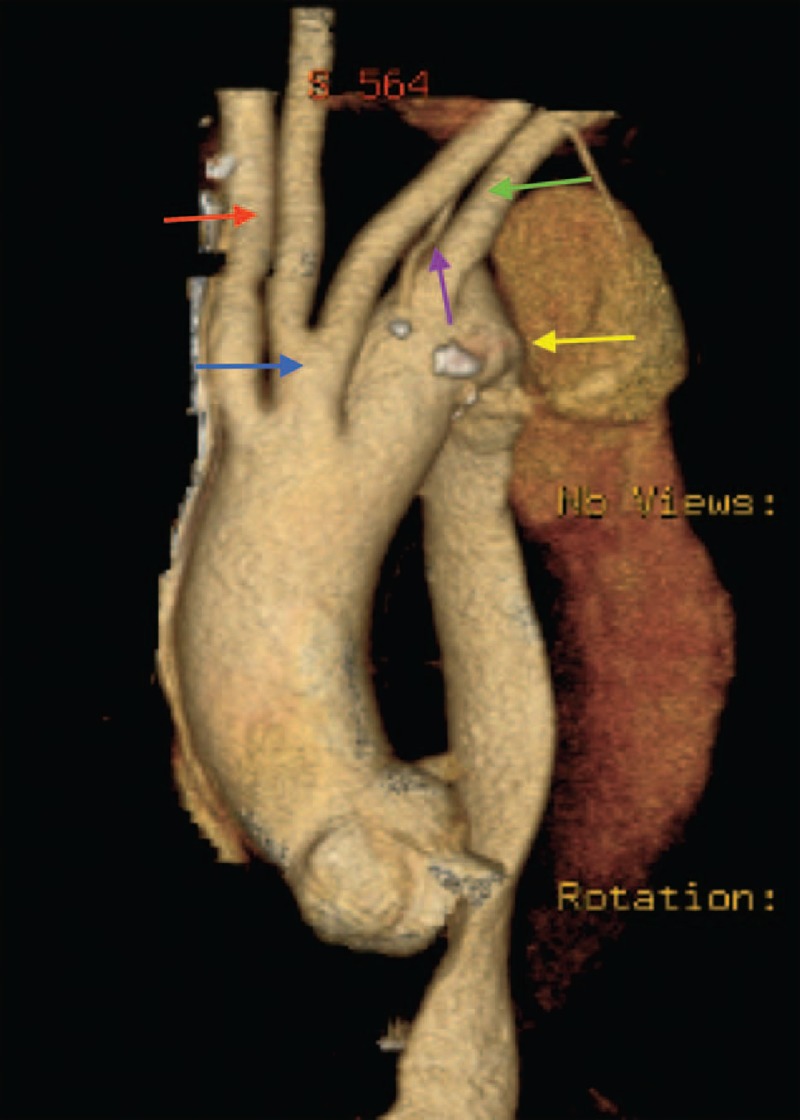
CT scan image (volume rendered). LSCVS and Stanford B aortic dissection. (Red arrow, right subclavian artery; blue arrow, common trunk of the carotid arteries; purple arrow, vertebral artery with direct origin of the arch; green arrow, left subclavian artery; yellow arrow, dissection.) CT = computed tomography, LSCVS = left aortic arch with right subclavian artery, common origin of carotid arteries, vertebral and left subclavian arteries.

**FIGURE 6:**
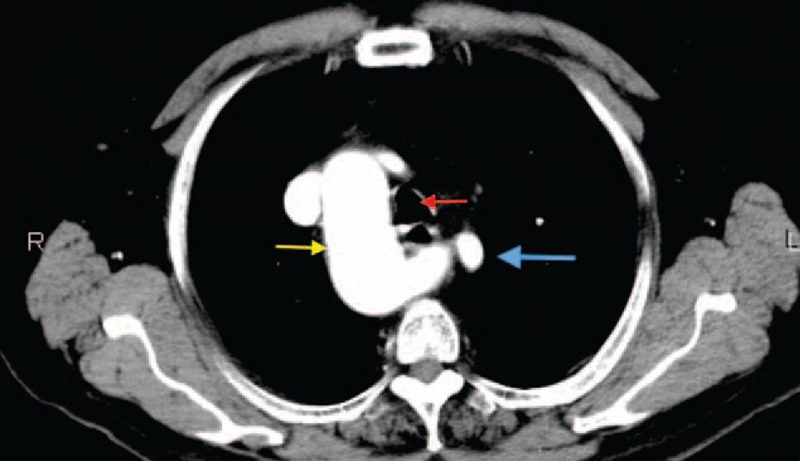
CT scan image. Axial view. RALS. (Blue arrow, aberrant left subclavian artery; red arrow, trachea; yellow arrow, right aortic arch.) CT = computed tomography, RALS = right aortic arch with aberrant left subclavian artery.

**FIGURE 7:**
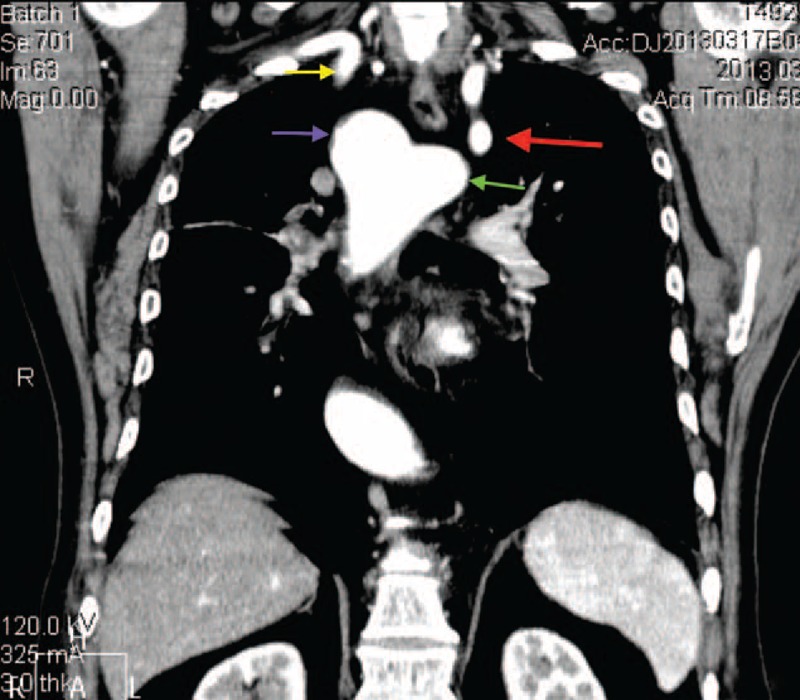
CT scan image. Anterior view. RALS. (Purple arrow, right aortic arch; red arrow, aberrant left subclavian artery; yellow arrow, right subclavian artery; green arrow, Kommerell diverticulum.) CT = computed tomography, RALS = right aortic arch with aberrant left subclavian artery.

The current study used 525 (n = 525) CT scans of patients with a diagnosis of TAD and 525 CT scans of patients without aortic disease. The study found 7 different patterns of the aortic arch in both groups of cases and controls (Table 1):

1. The aortic arch design is standard if it has 3 branches and is left sided in succession from right to left: brachiocephalic artery, left common carotid artery, and left subclavian artery.
2. Common origin of the brachiocephalic artery and left common carotid artery (bovine arch) (Figure 1).
3. Vertebral artery with direct origin from the aortic arch (vertebral pattern) (Figure 2).
4. Left aortic arch with right carotid artery, left carotid artery, left subclavian artery and aberrant right subclavian artery (LARS) (Figure 3).
5. Left aortic arch with common origin of the carotid arteries, left subclavian artery, and aberrant right subclavian artery (LCCARS) (Figure 4).
6. Left aortic arch with right subclavian artery, common origin of carotid arteries, vertebral and left subclavian arteries (LSCVS) (Figure 5).
7. Right aortic arch with aberrant left subclavian artery (RALS) (Figures 6 and 7).

**TABLE 1:**
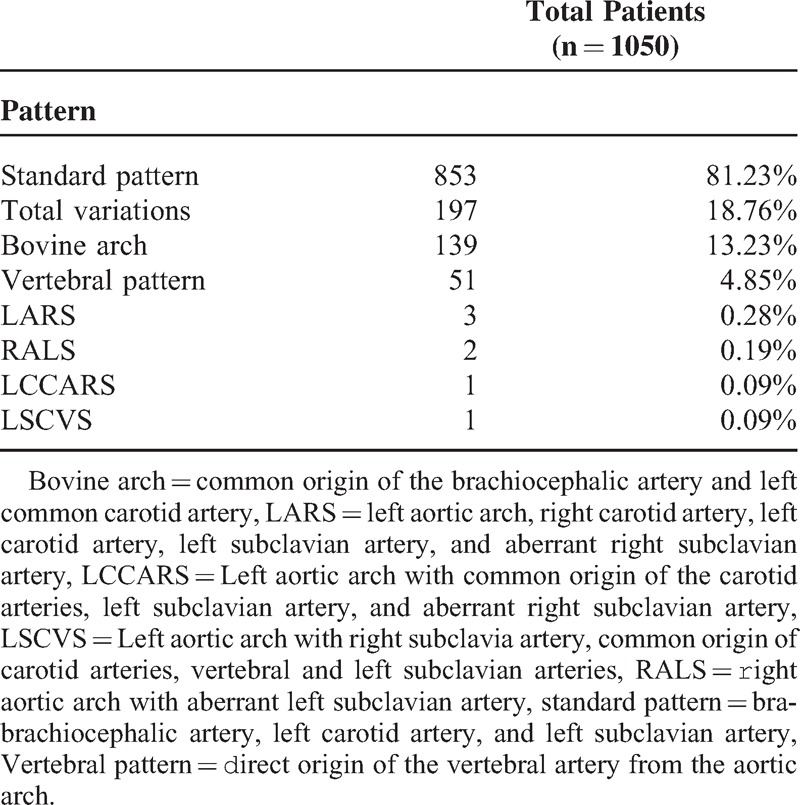
Aortic Arch Branching Patterns: General Statistic

### Anatomical Variations, Cases Versus Controls

Within the 525 patients with TAD 85 (16.19%) anatomic variations were observed, while the control group showed 112 variations (21.33%); *P* = 0.033. The most common anatomical variant was the bovine arch found in 62 (11.80%) cases of TAD compared with 77 (14.66%) in the control group; *P* = 0.172. The variant defined as the vertebral artery derived directly from the aortic arch was the second most frequent variation in patients with TAD and the control group; *P* = 0.114. The other anatomic variations showed frequencies less than 0.5% in both groups (Table 2).

**TABLE 2:**
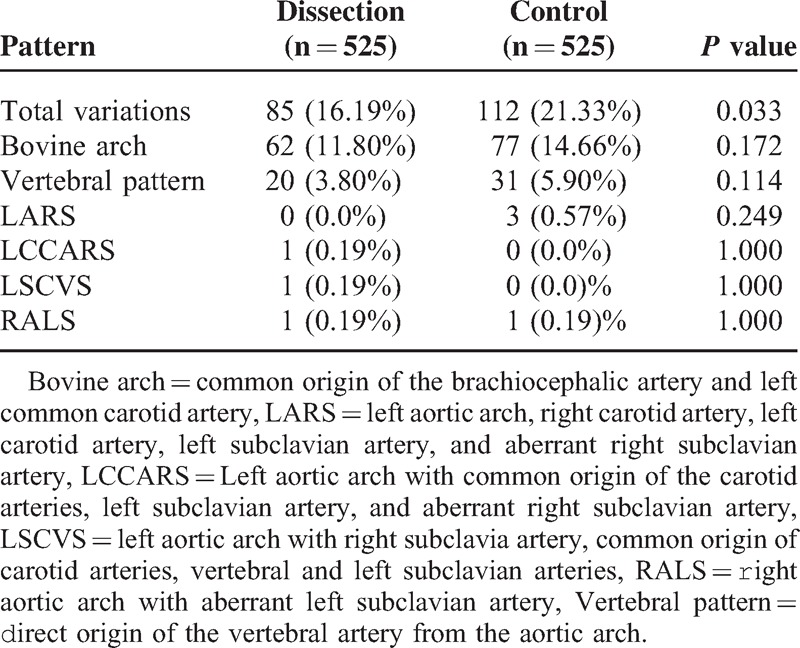
Aortic Arch Branching Patterns: Cases and controls

## AORTIC DISSECTION

The study showed 174 (n = 174) patients with Stanford A aortic dissection and 351 (n = 351) patients with Stanford B aortic dissection. In the Stanford A group were observed 25 (14.32%) anatomical variations and 60 (17.09%) in the Stanford B group; *P* = 0.425. The patients with Stanford A dissection presented the pattern of bovine arch in 23 (13.21%) of 174 cases, in contrast to the patients with Stanford B dissection, who showed it in 39 (11.11 %) of 351 cases; *P* = 0.481. The anatomical pattern defined as the vertebral artery of direct origin of the aortic arch was observed in 2 (1.14%) cases in the Stanford A group. The Stanford B group showed it in 18 cases (5.12%); *P* = 0.025.

LCCARS, LSCVS, and RALS variations were observed only in the Stanford B group (Table 3).

**TABLE 3:**
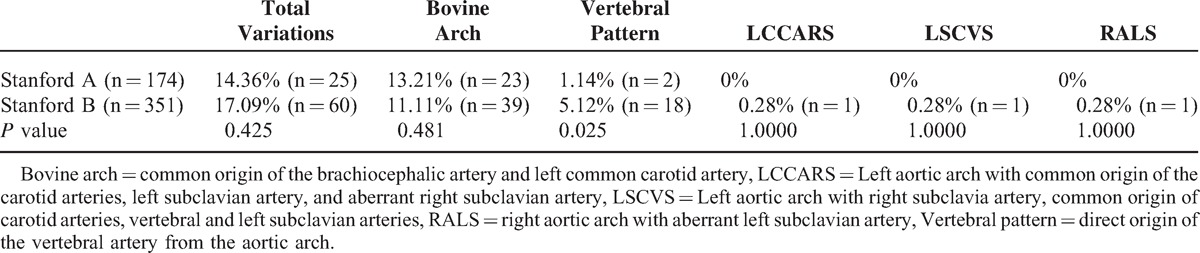
Arch Patterns by Location of Aortic Dissection

### Age

The patient series has 4 subgroups by age: less than 40 years old, between 41 and 60, between 61 and 79, and over 80. The patients without aortic disease in the subgroups from 41 to 60 years and older than 80 years presented more anatomical variations than the patients with TAD (*P* = 0.050; *P* = 0.030, respectively) (Table 4).

**TABLE 4:**
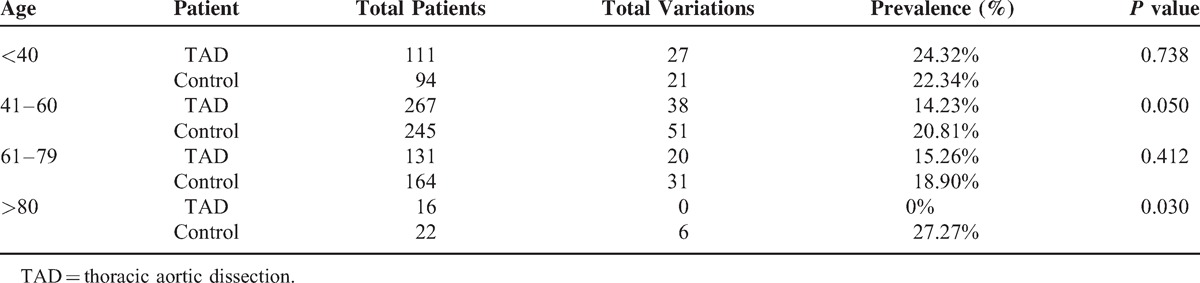
Arch Patterns by Age

### Gender

Within the 374 men with TAD, the study found 273 (72.9%) cases of Stanford B aortic dissection and 101 (27.0%) cases of Stanford A dissection. In the group of 151 women with TAD, 78 (51.65%) cases of Stanford B aortic dissection and 73 (48.34%) cases of Stanford A dissection (*P* = 0.001) were observed. The anatomical variations of the aortic arch with TAD occurred in 14.97% of men and 19.20% of women. The control groups showed it in 21.67% and 20.92%; *P* = 0.026, *P* = 0.681, respectively. The pattern of bovine arch was more frequent in both genders in the control group than the cases, without a statistically significative difference. The pattern of vertebral artery with direct origin of the aortic arch was less frequent among men with TAD than in the control group (*P* = 0.016), with similar findings in women (*P* = 0.639) (Table 5).

**TABLE 5:**
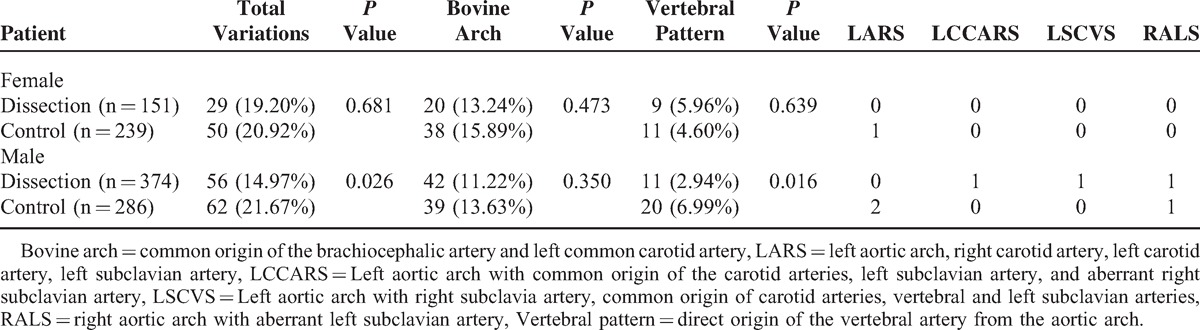
Arch Patterns by Gender

## DISSCUSION

The accurate information about the aorta arch branches variations in the different genetic backgrounds will help cardiovascular surgeons make decisions about the appropriate clinic treatment.

In Western studies, the standard pattern of the aortic arch occurs in approximately 80% of people.^13,14^ The bovine arch is the second most common design with 6.7% to 20% of patients without aortic aneurysm or dissection.^13,14,15,17,19,20^ The third most common is the vertebral pattern in 4% to 6% of the general population,^11,14,21,22^ which is consistent with our observations.

Our study of Asian patients showed a different pattern from the Western studies relating to racial differences in the anatomic variations of the aortic arch with dissection.

A lower frequency of anatomic variations was seen in this Chinese series than the patients studied by Wanamaker et al.^14^ The author found a higher frequency of aortic arch variations in African-American patients with TAD. He showed an anatomic variation incidence of 56% in this race compared with 32% of the patients without TAD. His study also found that whites with TAD have an incidence of 32%, compared with 18% of the patients without aortic dissection.

Anatomic studies of the aortic arch in other races show similar patterns that our survey. Ogeng’o et al^16^ in a Kenyan study of 113 aortic arches observed an incidence of 67.3% of the standard pattern. In line with our findings also is the cadaveric Korean study of Shin et al^23^ that found a frequency of 16% of aortic arch anatomic variations in patients without TAD.

In regard to the anatomical variant of the bovine arch, our study found 11.80% of the cases with TAD compared to 14.66% in the control group, whereas Wanamaker et al^14^ found that white patients had an incidence of 27.8% and African-Americans of 55.5% compared with 13.1% and 32% in the control groups, respectively. The Asiatic research of Shin et al^23^ demonstrated that 13.20% of the studied Korean corpses without aortic dissection presented the bovine arch. Ogeng’o et al^16^ from Africa encountered a rate of 25.7% of this pattern in black Kenyan cadavers without aortic dissection. The presence of the bovine arch in type B aortic dissection has been associated with risk of retrograde dissection during or after thoracic endovascular aortic repair (TEVAR). The occurrence ranges from 1% to 3%.^24,25^ The cause of this observation is possibly due to disorders of the aortic wall produced by changes in the extracellular matrix during the migration of the cells of the neural crest.^26^ In addition, the hemodynamic changes in the ascending aorta after the placement of an endoprosthesis could facilitate the retrograde dissection through the marginalization of the blood flow from the aortic valve and the changing pattern of stress of blood flow in aortic wall.^27,28^ To investigate those mechanisms would be interesting to establish histological and hemodynamic evaluations of aortas with bovine arch.

The incidence of the vertebral pattern varies between 0.79% described in the conventional angiographic study by Natsis et al^13^ and 6.1% found by Berko et al.^29^ Our study found that the second most frequent anatomical pattern was the vertebral pattern. This variant presented a frequency of 3.80% in the patients with TAD and 5.90% in the control group. These results are in line with what has been observed by Shin,^23^ who demonstrated this variation in 4.84% of the corpses of his Korean study. Similarly, Bhatia et al^30^ from South Australia, and Ogeng’o et al^16^ from Africa found rates of 7% and 6.7% of this pattern, respectively. From the surgical point of view, the preoperative diagnosis of the presence of a vertebral artery that originates directly from the arch is important, since it allows the planning of the method of cerebral protection during the procedure of reconstruction of the aortic arch.^19^ The presence of this artery is not associated with clinical symptoms. However, its pervasiveness has been associated with a risk of spontaneous vertebral artery dissection.^31^ The indications for revascularization of this artery could be the dominance of its flow; absence, hypoplasia, atresia, or stenosis of the right vertebral artery; and incomplete vertebrobasilar system.^32^ Misdiagnosis of the presence of this variation may produce mistakes, such as occlusion or endoleaks during endovascular treatment of type B aortic dissection or difficulties during surgical approach of the aortic arch branches in type A dissection. The current study observed a statistically significant relationship between the anatomical variant of 4 branches, defined as vertebral artery of direct origin of the aortic arch and the Stanford B aortic dissection. This finding may indicate that the geometry of the aortic arch would define the site of the first intimal tear of aortic dissection. Confirmation of this relevant information requires hemodynamic studies.

Freed et al^33^ described a prevalence of 0.4% to 2% of the LARS, which was also reflected in our study. The angle of the LARS may have a relationship with weakening of the aortic wall, which could increase the risk of TAD.^34^ The prevalence of aberrant right subclavian artery is higher in patients with right aortic arch compared with left aortic arch.^35^

There exist few reported cases of persistence of the common carotid trunk along with the presence of the vertebral artery of direct origin in the aortic arch, and the same way the presence of ARS.^36–40^ Our series observed a frequency of 0.28% of every anatomical variant with these characteristics.

Wang et al^41^ described the trend of early onset of the Stanford A dissection in 47.95 years of age and 53.37 years of age in the Stanford B dissection, which is consistent with what was observed in our study and could be related to the high levels of smoking among young people and the high levels of pollution in China.^42,43^ The International Registry of Aortic Dissection (IRAD) demonstrates a mean age of 63 years old for Western patients with Stanford A aortic dissection.^44^

Wang and collaborators^45^ have observed a statistically significant difference with respect to gender and dissection of the aorta. His finding is in line with our study that showed a higher incidence of disease in the male gender than in the female gender. Our finding of the gender difference in the aortic dissection is also consistent with what was described by LeMaire and Russell^2^ in the Western population.

The Chinese epidemiologic information about the aortic dissection is scarce due to the absence of national population-based studies. Therefore, the real incidence and mortality of this disease are still unknown. There are some studies based on hospital data, such as the study of Wang et al^45^ that performed a retrospective analysis of patients of 15 cardiovascular centers. This study gives insights into the epidemiological characteristics of aortic disease in China but lacks a sufficient number of patients to extrapolate results to the general population of China. The authors evaluated the clinical features of acute aortic dissection and compared them with data from the IRAD. Interestingly, they described a lower frequency of hypertension in Chinese patients than in patients of the Western series. Hypertension and smoking are considered common factors for aortic dissection due to their deleterious effect on the composition of the arterial wall. These factors produce smooth muscle necrosis and fibrosis of elastic structures of the aortic wall, creating a cradle for dissection. This finding may be related to genetic polymorphisms that affect the regulation of vascular tone on the Asian race. Santulli et al^46^ described that the deletion of the *CaMK4* gene is a factor associated with the presence of hypertension in Western patients. Probably, the Asian patients have some genetic polymorphism, such as those described by Santulli et al^46^ The genetic background might explain the results obtained by Wang,^41^ Wang,^45^ and our findings.

## LIMITATIONS OF THE STUDY

The retrospective design was the major limitation of the current study. Another limitation was that the study was completed at a single center. It would be important to make a multiethnic analysis about aortic anatomical patterns, since according to the results obtained in the current study, there is a divergence between what was observed in the Western series and this Chinese series. This knowledge could be a tool to guide the surgeon to deal with cases according to ethnic particularities.
